# On the Socio-Sexual Behaviour of the Extinct Ursid *Indarctos arctoides*: An Approach Based on Its Baculum Size and Morphology

**DOI:** 10.1371/journal.pone.0073711

**Published:** 2013-09-18

**Authors:** Juan Abella, Alberto Valenciano, Alejandro Pérez-Ramos, Plinio Montoya, Jorge Morales

**Affiliations:** 1 Institut Català de Paleontologia Miquel Crusafont, Universitat Autònoma de Barcelona. Edifici ICP, Campus de la UAB s/n, Barcelona, Spain; 2 Museo Nacional de Ciencias Naturales-CSIC, Madrid, Spain; 3 Departamento de Geología Sedimentaria y Cambio Medioambiental. Instituto de Geociencias (CSIC, UCM), Madrid, Spain; 4 Departamento de Paleontología, Facultad de Ciencias Geológicas UCM, Madrid, Spain; 5 Institut Cavanilles de Biodiversitat i Biologia Evolutiva, Universitat de València, Paterna, Spain; 6 Departament de Geologia, Àrea de Paleontologia, Universitat de València, Burjassot, Spain; University of Utah, United States of America

## Abstract

The fossil bacula, or *os penis*, constitutes a rare subject of study due to its scarcity in the fossil record. In the present paper we describe five bacula attributed to the bear *Indarctos arctoides* Depéret, 1895 from the Batallones-3 site (Madrid Basin, Spain). Both the length and morphology of this fossil bacula enabled us to make interpretative approaches to a series of ecological and ethological characters of this bear. Thus, we suggest that *I. arctoides* could have had prolonged periods of intromission and/or maintenance of intromission during the post-ejaculatory intervals, a multi-male mating system and large home range sizes and/or lower population density. Its size might also have helped females to choose from among the available males.

## Introduction

Batallones-3 is one of the nine Late Miocene (Late Vallesian) fossil vertebrate localities of the fossiliferous area of Cerro de los Batallones (Madrid Basin, Spain) [1], [2], [3], [4], [5]. Together with the lower levels of Batallones-1 and 2, it is considered to have acted as a natural trap, where carnivorans entered these pseudokarstic cavities and were trapped there [3], [6], [7], [8], [9]. In the sample recovered, almost 98% of the fossil mammal remains identified from Batallones-3 belong to carnivorans. Among the fossil species we found remains of the primitive bear *Indarctos arctoides* Déperet, 1895 with a total of 1690 bone elements, belonging to a minimum number of 16 individuals [6]. With several complete or nearly complete skeletons, including skulls, mandibles, vertebrae, scapulae, pelvis, limb bones and even the bacula, this sample is by far the most complete one of this species and probably of the genus.

The baculum or *os penis* is a non-appendicular bone found in the glans tissue, dorsal to the urethra, with its proximal end abutting the distal end of the *corpus cavernosum* [10], [11], [12] of members of five orders of mammals (specifically, some carnivorans, insectivorans, rodents, bats, and primates) [13], [14], [15]. The baculum has three developmentally distinct parts: a proximal portion derived from cartilage, a central portion derived from membranous bone, and a distal portion made up of non-lamellar bone. The proximal and central portions fuse and begin to ossify within five days of birth, but the distal portion does not ossify until puberty [16], [17], [18], [19].

Few papers have described the baculum of fossil species, and they have all been merely descriptive and have only compared it with the baculum of extant species [20], [21], [22].

### Description of the Baculum of *Indarctos arctoides* Déperet, 1895

The studied sample from Batallones-3 consists of five bacula or *os penis*, four of which belong to grown adults and the other one to a sub-adult or young adult, matching quite well with the previous sex determination of the sample from the site, 4–6 of which were males [6].

**Figure 1:**
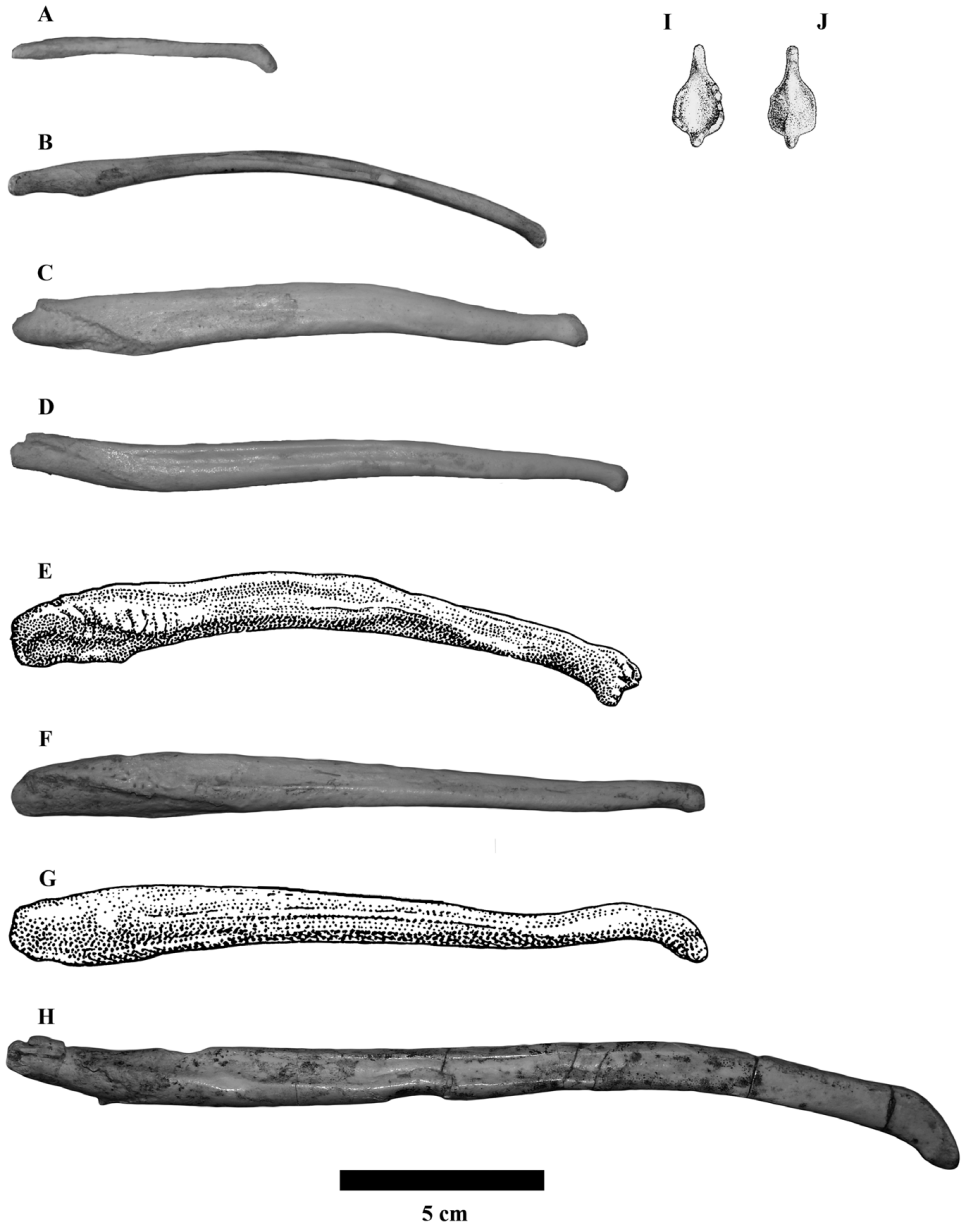
Modified from Didier 1950 and Davis 1964. Baculum in laterial view of: A Helarctos malayanus; B Ursus thibetanus; C Tremarctos ornatus; D Ursus americanus; E Melursus ursinus; F Ursus arctos; G Ursus maritimus; H Indarctos arctoides; I Ventral view of the Baculum of Ailuropoda melanoleuca; J Dorsal view of the baculum of Ailuropoda melanoleuca.

The baculum of *Indarctos arctoides* (Figure 1H and Supplementary Videos: Video S1–S5) is a slightly sigmoidal elongated bone. Its base (proximal part) is curved dorsally while the apex (distal part) is curved ventrally. It has a triangular section along its length, except at both ends, where the base is oval and the apex is sub-rectangular. It does not have a marked urethral groove, the ventral area therefore being flat except at the apex, where it has a small groove, and the distal zone, which appears somewhat concave. However, it has two longitudinal grooves on its dorsal part, which run along most of the surface from the rough area of the base to close to the apex. The proximal region has a very rough surface, which occupies most of the lateral side of the bone, while in the ventral area a small ridge-shaped structure is developed ventrally. On the dorsal side, the roughened area is not as obvious and is limited to the lateral areas, while the central surface is smooth along the dorsal part of the bone. The apex is completely preserved in three of the five bacula and its morphology is virtually identical in all three. It consists of a ventrally curved structure with a triangular-shaped longitudinal notch, which occupies the ventral part corresponding to the distal-most part of the urethral groove. Its lateral surface is quite flat, somewhat concave, especially in the specimen BAT-3**′**10.1062. Its dorsal area is rather oval and with a porous texture. Its central part is slightly sunken forming a slight concavity in BAT-3**′**10.1062, whereas in BAT-3**′**08.869 it is fairly flat. In the apical-most zone of the baculum, all these surfaces converge in a sub-rectangular tip that is projected ventrally.

The mean length of the bone is 225.26 mm, the shortest one measuring 191.9 mm and the longest 237.7 mm. However, the shortest baculum was associated *in situ* with a sub-adult skeleton, and we therefore consider it to be an undeveloped baculum, which would have been larger with age [15]. The ontogeny of the baculum has been studied in bears and mustelids [15], [23], [24]. The length of the baculum in polar bears, for example, almost reaches its full length before the bear is 10 years of age, and its growth is most rapid between the first and second years of its life [15].

Adult mean length can therefore be established at 233.6 mm. (without taking into account the length of the shorter baculum).

## Comparison

The bacula of most extant species of Ursidae species are relatively long for their body size. However, the baculum of the giant panda (*Ailuropoda melanoleuca*), is the only reduced *os penis* of the family [25], [26], [27].

The bacula of *Indarctos* from Batallones-3 has been compared to the homologues of all of the extant bear species (Figure 1A–J). Its morphology matches perfectly with that of this family, with an almost straight overall lateral outline, a longitudinal groove on each side of its dorsal region and a distal end slightly curved ventrally. It is easily distinguished from the canid and amphicyonid bacula by the absence of a well-developed urethral groove [21] and from the mustelid bacula by the shape of the lateral outline [22].

When compared to the bacula of the extant bears at a more detailed scale, the os penis of *Indarctos arctoides* is relatively more elongated than that of the other species. Its shape is slightly more sigmoidal and its distal tip is relatively better developed; only the baculum of *Helarctos malayanus* has developed an ossified tip at its distal end (Figure 1A). Furthermore, the apical anatomy has been observed to be intra-specifically diverse in the polar bears, and its apical growth continues into late life in at least some species [15], [28], [29], [30], [31], [32], [33]. In other groups, such as canidae, the tip is cartilaginous and not completely ossified throughout its life cycle [34].

Although *Indarctos* is now considered to be a member of the subfamily Ailuropodinae [35] its baculum is one of the longest *os penis*, relative to its body size, to be found in the bear family. *Indarctos arctoides* must have been a large bear similar in size to the European brown bear; it had a mean estimated body mass of 202.16 kg; it presented evident sexual dimorphism with a body mass estimated at 265.74 kg for males and 137.30 kg for females [6]. Their bacula could also be expected to be large.

Thus, only taking into account the lengths of the adult bacula from Batallones-3, mean sample length exceeds the largest size of the homologues of both polar and grizzly bears, the largest extant species of Ursidae [15], [25], [36]. Bacular length in adult polar bears, for example, was reported as 168 mm, 168–194 mm and 151–222 mm by Didier ([25]; *n* = 1), Lønø ([36]; *n* = 15), and *Dyck et al*. ([15]; n = 871) respectively.

## Discussion

The relatively long baculum of *Indarctos arctoides* might have been related to several socio-sexual behaviour patterns of this species, such as a prolonged, single-intromission copulatory pattern and/or maintenance of intromission during the post-ejaculatory interval [37], [38], [39], [40].

It has been suggested that larger bacula, together with a more complex genital morphology are observed in species with multi-male mating systems [37], [38], [41], [42], while smaller ones are more usual in polygynous species, suggesting that multi-male mating may be the female’s strategy to evaluate mate choice when mate availability is limited or sequential [43]. There is empirical evidence of post-copulatory selection occurs in species that adopt multi-male matings and in which multiple paternity is observed (e.g. *Ursus americanus* [44]). Furthermore, the baculum may also play a more significant role in monogamous (such as in Canidae) and multi-male mating systems (such as in Mustelidae; [45]) as an indicator of genetic quality in males [46], and is used by females as a way of assessing male quality when mate encounters are disjunct temporally and spatially [47].

Another factor likely to vary among mammalian groups is the degree to which the site of ejaculate deposition in the female tract is critical to fertilization success [48], [49], longer bacula could ensure that sperm and/or copulatory plugs are optimally positioned in the female reproductive tract (the position of the latter being critical with regard to maximizing sperm transport) [50] or could facilitate the removal of previously deposited plugs [51], [52]. Several studies have also led some researchers to link longer bacula with delayed implantation/embryonic diapause and multi-male mating systems, as a response to environments with acute unfavourable seasons [42].

When comparing baculum length to body size in the Ursidae, it can be observed that larger species such as *Ursus maritimus* and *U. arctos* have relatively shorter bacula than smaller species such as *U. americanus*, *Helarctos malayanus* or *U. thibetanus* (Table 1). It seems that the *os penis* is relatively shorter in the more sexually dimorphic species, which have a more polygynous mating system and whose competition for the females is intense [65], [66], [67]. However, in *Indarctos arctoides*, despite being a large-sized and highly dimorphic species [6] the length of the baculum is relatively long compared to other bear species of similar size, probably due to the fact that this feature is likely sexually selected.

**Table 1:** Measurements of the baculum length (in mm) and aproximate worldwide average male body size (in Kg) for the eight extant species of Ursidae and the estimated average size calculated for the males of *Indarctos arctoides* from Batallone-3.

Species	Baculumlength	Male bodysize	BL/BSindex
*Helarctos malayanus*	52	60	0,87
*Melursus ursinus*	151	110	1,37
*Ailuropoda melanoleuca*	20	120	0,17
*Tremarctos Ornatus*	104,7	127,5	0,82
*Ursus americanus*	137	140	0,98
*Ursus thibetanus*	118,25	150	0,79
*Ursus arctos*	133,8	253	0,53
***Indarctos arctoides***	**233,6**	**265,74**	**0,88**
*Ursus maritimus*	186,5	500	0,37

The index “baculum length divided by body size” has also been calculated to compare relative sizes. The body size values have been obtained from references [26], [53]–[64].

The information on the carnivoran association of Batallones-3 is yet to be fully completed, since only several species have been studied [3], [6], [7], [8], [9]. However, there are many papers on the association of Batallones-1, which can be used to make comparisons among the carnivorans that these two sites have in common [68], [69], [70], [71], [72], [73], [74], [75], [76], [77], [78]. According to the abovementioned papers, some kind of ecological segregations may have enabled all these carnivorans to reduce the frequency of encounters. Furthermore, in Batallones-3, these ecological segregations must have been more acute, reducing the frequency of encounters among many large-sized predators, since there are at least four large species weighing over 100 kg, two more than in Batallones-1 [6], [7].

### Inferences Regarding the Socio-sexual Behaviour of *Indarctos arctoides*

The length of the baculum could be related to several sexual, social and ecological behaviour patterns. However, these are either ecological and/or ethological characteristics of this fossil species and are therefore not possible to verify.

Therefore, and based on the features of the baculum of *Indarctos*, we suggest that this species may have had:

1. A prolonged period of intromission and/or maintenance of intromission during the post-ejaculatory interval. The baculum may have acted as a physical support during the penetration and served to maintain vaginal opening during the ejaculatory interval. In extant species a positive relation between the length of the bacula and the time and maintenance of intromission has been described.
2. Multi-male mating system. Possibly, the home range of several males overlapped with those of one or more females, enabling fecundation to be more successful in these infrequent sexual encounters.
3. The evident sexual dimorphism present in the body size of *Indarctos arctoides* may also have been a characteristic to be considered. Contrary to the relatively shorter baculum found in bear species presenting strong sexual dimorphism, the baculum of *I. arctoides* was comparatively long. This feature, could have have caused the females to exhibit preferential mating among the available males, as seen in extant species.
4. *Indarctos arctoides* could have had large home range sizes and/or a lower population density. In this specific case, the presence of patched forests separated by more open environments may have caused the populations of this bear to be less, thus giving rise to fewer encounters among the individuals.

## Supporting Information

Video S1 **Video of the baculum BAT-3′08.869.**
(AVI)

Video S2 **Video of the baculum BAT-3′10.428.**
(AVI)

Video S3 **Video of the baculum BAT-3′10.1062.**
(AVI)

Video S4 **Video of the baculum BAT-3′12.815.**
(AVI)

Video S5 **Video of the baculum BAT-3′12.2298.**
(AVI)
